# Association of a miRNA-137 Polymorphism with Schizophrenia in a Southern Chinese Han Population

**DOI:** 10.1155/2014/751267

**Published:** 2014-08-27

**Authors:** Guoda Ma, Jingwen Yin, Jiawu Fu, Xudong Luo, Haihong Zhou, Hua Tao, Lili Cui, You Li, Zhixiong Lin, Bin Zhao, Zheng Li, Juda Lin, Keshen Li

**Affiliations:** ^1^Institute of Neurology, Guangdong Medical College, Zhanjiang 524001, China; ^2^Department of Psychiatry, Affiliated Hospital of Guangdong Medical College, Zhanjiang 524001, China; ^3^Unit on Synapse Development and Plasticity, National Institute of Mental Health, National Institutes of Health, Bethesda, MD 20892, USA

## Abstract

Both genome wide association study (GWAS) and biochemical studies of Caucasian populations indicate a robust association between the miR-137 genetic variant rs1625579 and schizophrenia, but inconsistent results have been reported. To assay the association between this variant and schizophrenia, we genotyped 611 schizophrenic patients from Southern Chinese Han population for the risk single nucleotide polymorphism (SNP) rs1625579 using the SNaPshot technique and compared the clinical profiles of different genotypes. Additionally, a meta-analysis was performed using the combined sample groups from five case-control publications and the present study. Both the genotype and allele distributions of the rs1625579 SNP were significantly different between patients and controls (*P* = 0.036 and 0.026, SNP). TT genotype carriers showed slightly lower Brief Assessment of Cognition in Schizophrenia- (BACS-) derived working memory performance than G carriers (15.58 ± 9.56 versus 19.71 ± 8.18, *P* = 0.045). In the meta-analysis, we observed a significant association between rs1625579 and schizophrenia under different genetic models (all *P* < 0.05). The results of our study and meta-analysis provide convincing evidence that rs1625579 is significantly associated with schizophrenia. Furthermore, the miR-137 polymorphism influences the working memory performance of schizophrenic patients in a Chinese Han population.

## 1. Introduction

miRNAs are small, noncoding RNAs that regulate gene expression posttranscriptionally [1]. Over half of the miRNAs identified thus far are highly or exclusively expressed in the brain, where they act as integral regulators of neuronal genes involved in the function, plasticity, and development of the brain [2, 3]. miRNAs and their regulatory networks are thought to contribute to the complex processes underlying the etiology and pathophysiology of psychiatric disorders including schizophrenia [4–6].

Hsa-miR-137, as a brain-enriched micro-RNA, has important roles during neurogenesis, including the proliferation and differentiation of neural stem cells, the regulation of dendritic morphogenesis, and synaptic plasticity [2, 7, 8]. Furthermore, miR-137 is a candidate gene for schizophrenia susceptibility and was identified by a recent mega-GWAS analysis in the sample of European ancestry conducted by the Psychiatric GWAS Consortium (PGC) [9], where a polymorphism (rs1625579) located in the primary transcript of hsa-miR-137 had the strongest genetic signal associated with schizophrenia. Moreover, four other schizophrenia associated genes (TCF4, CACNA1C, CSMD1, and C10orf26) identified in that study contain predicted miR-137 binding sites [10], suggesting that the interplay between miR-137 and its target genes could be involved in the etiology of schizophrenia [11–13].

Since the publication of the 2011 GWAS study [9], many subsequent independent studies have suggested a significant heterogeneity for the rs1625579 polymorphism of miR-137 in schizophrenia across ethnicities [14–18]. Recently, Guan et al. [19] also found that the rs1625579 locus was associated with schizophrenia risk in a Northern Chinese cohort. However, they did not report the effects of miR-137 on certain traits of schizophrenia, such as age at onset, clinical symptoms, and cognitive functions. In the present study, we conducted a hospital-based case-control study to address this question in a Southern Chinese Han population and confirmed our findings in a meta-analysis with previously published populations. Moreover, we also evaluated the association between miR-137 polymorphism and the clinical characteristics of schizophrenia.

## 2. Subjects and Methods

### 2.1. Subjects

We recruited 611 schizophrenic patients and 628 healthy controls from the affiliated hospital of Guangdong Medical College. All patients underwent a standardized battery of examinations, including family history, extensive drug and alcohol assessment, physical and neurological examination, and laboratory tests to exclude substance-induced psychotic disorders or psychosis caused by general medical condition. The diagnosis was assigned independently by two experienced senior psychiatrists based on the Diagnostic and Statistical Manual of Mental Disorders IV (DSM-IV). Psychotic symptoms were evaluated in accordance with structured interviews for the Positive and Negative Symptom Scale (PANSS). The healthy controls were recruited from the Medical Center of the affiliated hospital of Guangdong Medical College. Based on unstructured interviews, participants who had a personal or family history of major psychiatric disorders, serious somatic illnesses, and substance abuse were excluded. The patients and controls were comparable in age and sex distribution (Table 1). The study protocol was approved by the ethics committee of Guangdong Medical College, and written informed consent was obtained from the participants and/or direct relatives.

### 2.2. DNA Extraction and Genotyping

DNA from blood samples was extracted using the Blood DNA Kit (Tiangen, China). SNP rs1625579 was genotyped using the SNaPshot technique with polymerase chain reaction (PCR) primers 5′-TTTAGTCAACATTTGCATTTGGAAGC-3′ and 5′-CAACAGATTCCAAAGGTCTCTAGTGTGC-3′. PCR was performed in a 20 *μ*L reaction composed of 1 × GC Buffer, 3.0 mM Mg^2+^, 0.3 mM dNTPs, 1 U of HotStarTaq polymerase (Qiagen, Germany), 1 *μ*L of template DNA (each at a final concentration of 5–10 ng/*μ*L), and 2 *μ*L of PCR primers. Amplification was performed with the following program: 2 min at 95°C, followed by 11 cycles of 94°C for 20 s, 65°C ± 0.5°C/cycle for 40 s, and 72°C for 1.5 min, and then 24 cycles consisting of 94°C for 20 s, 59°C for 30 s, and 72°C for 1.5 min, with a final extension step at 72°C for 2 min. The extension primer was 5′-TTTTTTTTTTTTTTTTTTTTTAAACAAGGGAAATGTTAATCACAATTA-3′. The single-nucleotide primer extension reaction was conducted using the ABI PRISM SNaPshot Multiplex Kit Protocol (Applied Biosystems, USA). Then, 0.5 *μ*L of the purified single base extension products was mixed with 9 *μ*L of Hi-Di formamide and 0.5 *μ*L of GeneScan-LIZ 120 internal size standard. After a denaturing step for 2 min at 95°C followed by cooling to 5°C, the mixture was further analyzed by capillary electrophoresis using an ABI Prism 3130xl Genetic Analyzer (Applied Biosystems, USA). The data were analyzed using GeneMapper 4.1 (Applied Biosystems, USA).

### 2.3. Neurocognitive Function Assessment

The Brief Assessment of Cognition in Schizophrenia (BACS) assesses the different aspects of cognition found to be important for cognitive impairment in patients with schizophrenia: (1) verbal memory measured by List Learning; (2) working memory measured by the Digit Sequencing Task; (3) motor speed measured by the Token Motor Task; (4) verbal fluency assessed using the Category Instances and Controlled Oral Word Association Test; (5) reasoning and problem solving assessed using the Tower of London; and (6) attention and processing speed assessed using Symbol Coding. The battery of tests requires approximately 30–35 minutes to complete in patients with schizophrenia and is scored by two psychologists. Further details on the BACS assessment were reported elsewhere by Keefe et al. [20, 21].

**Figure 1:**
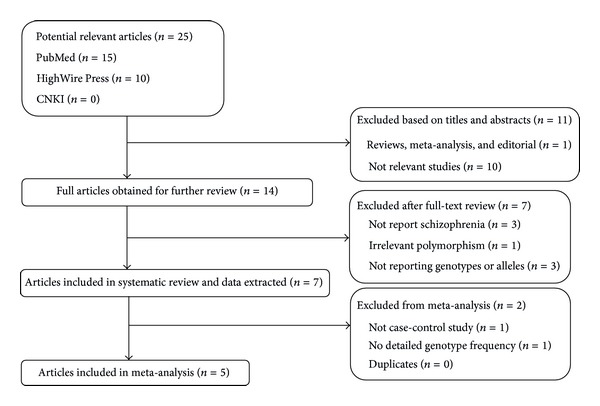
Flow diagram of the article selection process.

*Meta-Analysis*. The references included in the analysis were searched using Pubmed (http://www.ncbi.nlm.nih.gov/pubmed), HighWire Press (http://highwire.stanford.edu), China National Knowledge Infrastructure (CNKI: http://www.cnki.net), and Google Scholar (http://scholar.google.com) without any language limitations, focusing on the keywords “schizophrenia” and “miR-137” or “microRNA-137.” The literature search was updated on May 10th, 2014. The criteria for inclusion were as follows: association studies written in English; diagnosis of schizophrenia according to standardized criteria, such as DSM or ICD-10; case-control studies investigating rs1625579 genotype and allele frequencies; available genotyping method and sufficient data to calculate the odds ratio (OR) with 95% confidence intervals (CI) and *P* values. The following were exclusion criteria: reviews or abstracts; studies which were not relevant to miR-137 gene rs1625579 polymorphism or schizophrenia; studies which did not report genotype frequencies. The flow of the article selection process was shown in Figure 1. In total, five independent case-control studies were selected [14, 16–19]. Then the following information was extracted from each study: the name of first author; the year of publication; the ethnicity of the study population; the numbers and frequencies of rs1625579 polymorphism genotypes in cases and controls; the OR with 95% CI or if not provided, calculated the OR and 95% CI. The quality evaluation was performed with the Newcastle-Ottawa Quality Assessment Scale for case-control studies.

We evaluated the genetic heterogeneity among the studies included using Cochran's *Q* test, which approximately follows a *χ* ^2^ distribution with *k* − 1 degrees of freedom (*k* stands for the number of studies for analysis). Another statistic *I* ^2^ = ((*Q* − (*k* − 1))/*Q*) × 100% was also used. *I* ^2^ ranges from 0 to 100% [22]. Low, moderate, large, and extreme heterogeneity corresponded to 0–25%, 25–50%, 50–75%, and 75–100%, respectively [22]. We defined the significant heterogeneity with *P* < 0.01 and *I* ^2^ > 50%.

All statistical tests for heterogeneity and meta-analysis were performed using RevMan (v.5.1) software (http://ims.cochrane.org/revman/download). If there was no significant heterogeneity among the total of six studies, including the present study (total of 3,133 patients and 2,791 controls), OR with corresponding 95% CI was calculated by the fixed effect model (Mantel-Haenszel); otherwise the OR was calculated by random-effect model (DerSimonian-Laird). *Z* test was used to determine the significance of OR.

Four genetic models were used in our meta-analysis, which can be described as follows: the dominant model (TT + GT versus GG), the recessive model (TT versus GT + GG), the additive model (TT versus GG), and the allele model (T allele versus G allele). In order to validate the stability of our results, sensitivity analysis for the overall effect was performed by omitting one case-control study at a time. Additionally, the publication bias was investigated with Egger's test and Begg's test using Stata 12.0 software.

## 3. Statistical Analysis

Hardy-Weinberg equilibrium was tested using Pearson's chi-squared test. Given the low frequency of the GG genotype in both schizophrenic patients (0.5%) and controls (1.0%), these cases were combined with those having the GT genotype to form the “G-allele carrier” group for all statistical tests. Statistical analyses comparing allelic and genotypic distributions were performed using Pearson's chi-squared test or Student's *t*-test. Generalized ORs with 95% CIs of the alleles were calculated. Descriptive variables were presented as the mean ± standard deviation (SD). *P* < 0.05 was considered significant for all statistical tests. The statistical analysis was performed using SPSS19.0 software, and the power calculation was performed using QUANTO 1.2 software (http://biostats.usc.edu/software).

## 4. Results

The distribution of the miR-137 rs1625579 polymorphism in our cohort is described in Table 2. Both the schizophrenia and control populations were in Hardy-Weinberg equilibrium (all *P* > 0.05). The frequency of the TT, TG, and GG genotypes was 90.02% (*n* = 550), 9.66% (*n* = 59), and 0.33% (*n* = 2), respectively, in the schizophrenic patients, and 86.15% (*n* = 541), 13.06% (*n* = 82) and 0.80% (*n* = 5), respectively, in the controls. Because of the high prevalence of the presumed risk allele (T), we conducted the analysis according to the presence of two copies of the risk allele (TT) or only one or no copies (GT or GG, resp.). A significant association was observed between the case and control groups for genotype (*χ* ^2^ = 4.409, *P* = 0.036) and allele (*χ* ^2^ = 4.971, *P* = 0.026).

Power was calculated using a G allele frequency of 0.073. Our entire cohort had a power of 0.813 for detecting a genotype relative risk with an OR of 1.5 at the 0.05 level. Therefore, our finding could be statistically strong with an appropriate sample size.

When the samples were stratified according to sex, we did not find any significant difference in either the genotype (*χ* ^2^ = 2.275, *P* = 0.131 for male; *χ* ^2^ = 2.240, *P* = 0.135 for female) or allele (*χ* ^2^ = 2.562, *P* = 0.109 for male; *χ* ^2^ = 2.548, *P* = 0.110 for female) distributions. Additionally, we focused on the description of relevant clinical characteristics. No significant differences were observed with regard to age, disease duration, age at onset, family history, and PANSS clinical symptom scores (Table 3).

Neurocognitive functions were determined using BACS. The sample for neurocognitive testing consisted of 209 clinically stable patients. As shown in Table 3, we observed that the T allele for SNP rs1625579 was associated with lower digit sequencing scores (*t* = 2.016, *P* = 0.045). There was no significant difference in the scores of the other BACS items.

**Figure 2:**
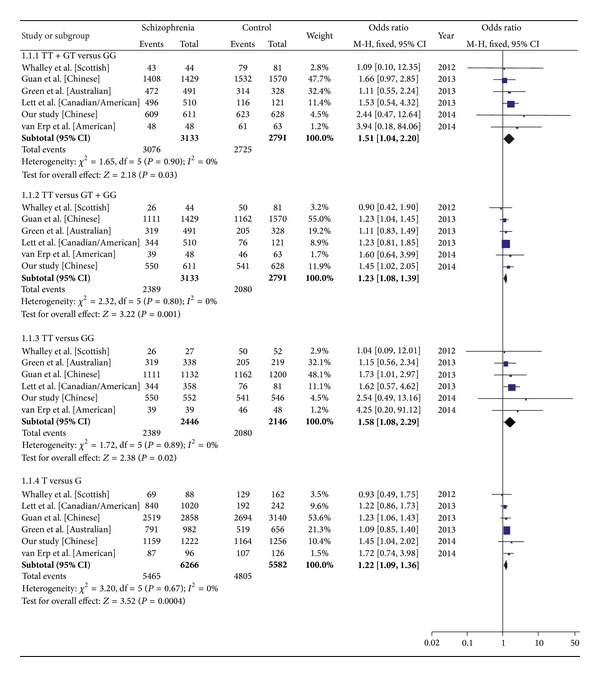
Forest plot of the meta-analysis for an association between the rs1625579 polymorphism and schizophrenia using dominant, recessive, additive, and allele models. 1.1.1 dominant model (TT + GT versus GG); 1.1.2 recessive model (TT versus GT + GG); 1.1.3 additive model (TT versus GG); 1.1.4 allele model (T versus G); M-H: Mantel-Haenszel, Fixed: fixed effect model; CI: confidence interval.

In the meta-analysis derived from five published case-control studies and the present association study, no significant heterogeneity was observed in the total analysis with different models (*I* ^2^ = 0.0% for all genetic models). Associations, however, were observed between miR-137 polymorphism and schizophrenia using a dominant model (*P* = 0.03, OR = 1.51, 95% CI: 1.04–2.20), recessive model (*P* = 0.001, OR = 1.23, 95% CI: 1.08–1.39), additive model (*P* = 0.02, OR = 1.58, 95% CI: 1.08–2.29), and allele model (*P* = 0.0004, OR = 1.22, 95% CI: 1.09–1.36) (Figure 2). There was no significant publication bias for the dominant model (Begg's test, *P* = 0.45, and Egger's test, *P* = 0.53), recessive model (Begg's test, *P* = 1.00, and Egger's test, *P* = 0.97), additive model (Begg's test, *P* = 0.45, and Egger's test, *P* = 0.56), or allele model (Begg's test, *P* = 1.00, and Egger's test, *P* = 0.83). The sensitivity analysis indicated that no single study qualitatively affected the summary risks (data not shown).

## 5. Discussion

The miR-137 gene was highly associated with schizophrenia in a recent GWAS analysis with an initial sample size of 21,856 and a replication sample size of 29,839 [9]. In two accompanying papers, five schizophrenia susceptibility genes including CSMD1, C10orf26, CACNA1C, TCF4, and ZNF804A were validated as miR-137 targets [10, 23], providing additional evidence for the involvement of miR-137 in schizophrenia. Along these lines, Wright et al. [24] recently used bioinformatic tools to examine putative targets of miR-137 and their potential contributions to neuronal functions. The top scoring pathways predicted by Wright et al. are related to long-term potentiation (LTP), ephrin receptor signaling, and axonal guidance, all of which are associated with learning, memory, and synaptic formation and are impaired in schizophrenia [25, 26]. Importantly, a recent postmortem study showed that miR-137 was downregulated in the dorsolateral prefrontal cortex of schizophrenic patients [27]. Together, these findings suggest that dysregulation of miR-137 may contribute to the pathology of schizophrenia.

In an attempt to replicate the GWAS findings in Asian subjects, we examined the miR-137 rs1625579 polymorphism in a Chinese Han cohort comprising 611 schizophrenic patients and 628 healthy controls. Significant differences in the genotype or allele distribution of the rs1625579 SNP were identified between schizophrenic patients and controls (*χ* ^2^ = 4.409, *P* = 0.036, and *χ* ^2^ = 4.971, *P* = 0.026, resp.). These results were replicated in a meta-analysis (*P* = 0.03, OR = 1.51, 95% CI: 1.04–2.20 for the dominant model; *P* = 0.001, OR = 1.23, 95% CI: 1.08–1.39 for the recessive model; *P* = 0.02, OR = 1.58, 95% CI: 1.08–2.29 for the additive model, and *P* = 0.0004, OR = 1.22, 95% CI: 1.09–1.36 for the allele model). The data demonstrate that the miR-137 polymorphism may affect susceptibility to schizophrenia under a recessive model for the T allele. This finding is in agreement with a GWAS [9] and a previous study in a northern Chinese cohort of Xi'an by Guan et al. [19]. In the present study, the frequency of the GG genotype was extremely low in schizophrenic patients and healthy controls (0.33% in the cases and 0.80% in the controls). Additionally, a lower frequency of the rs1625579 G-allele was detected in our cohort from Southern China (5.16% in the cases and 7.32% in the controls) than previously observed in American and Canadian (17.65% in the cases and 20.66% in the controls) [17], Australian (19.45% in the cases and 20.88% in the controls) [16], and Scottish (26.85% in the cases and 20.37% in the controls) [14] populations. These values reveal an ethnic heterogeneity in the distribution of the miR-137 polymorphism. In the same ethnicity, the G allele frequency of rs1625579 in our cohort from Zhanjiang, in Southern China, was also markedly lower than that in the Chinese cohort of Xi'an from a previous study [19] (11.9% in the cases and 14.2% in the controls) but similar to the level in the Chinese population in Beijing from the International HapMap Project (the minor allele frequencies = 0.085) (http://hapmap.ncbi.nlm.nih.gov). Therefore, genetic variation of miR-137 also exists among geographic groups within the Chinese population. However, our results and meta-analysis provide further support for the association between miR-137 polymorphism and schizophrenia even with the lower frequency of the G allele.

Our study demonstrated a weak but significant association between the TT genotype and lower scores in digit sequencing for working memory in the Chinese Han population, which is similar to the results of Cummings et al. [15], who showed that the polymorphism rs1625579 was also associated with cognitive deficits in schizophrenia, including working memory (assessed using the CANTAB Spatial Working Memory task) (*P* < 0.0001). In addition, overlapping microdeletion of the miR-137 locus in the chromosomal region 1p21.3 has been identified in patients with intellectual disabilities [28]. Therefore, we speculate that miR-137 is a key regulator in learning and memory processes. Considering that cognitive impairment is involved in the development of dysfunctional (neural, synaptic) connectivity between brain regions [29, 30], the lower performance of working memory associated with the TT genotype in our patients may be ascribed to inefficiency in neural functioning, which is regulated by miR-137 polymorphism. This viewpoint is supported by van Erp et al. [18] who found that lower working memory performance was related to higher left dorsolateral prefrontal cortex (DLPFC) activation in schizophrenic patients. Although there was no effect of genotype on working memory performance in schizophrenic patients, individuals with the rs1625579 TT genotype had significantly higher left DLPFC activation than those with the GG/GT genotypes during a working memory test. This working memory-associated DLPFC hyperactivation was interpreted as being consistent with neural inefficiency in carriers of the rs1625579 schizophrenia risk allele. This finding also showed that rs1625579 affected other neuroimaging phenotypes of schizophrenia. The T allele was associated with decreased hippocampal volume and reduced white matter integrity throughout the entire brain in the study by Lett et al. [17]. In our research, BACS assessment was only conducted on 209 samples, approximately 1/3 of the original cohort. Considering the limited sample size in this analysis, we cannot exclude the possibility that the statistical significance is due to the occurrence of the type I error. Given these possibilities, a replication study with lager sample size would be helpful in addressing this limitation and giving a more definitive view of the results reported in this present study.

We failed to find an association between rs1625579 and other clinical characteristics of schizophrenia, including age onset and PANSS scores. However, recent investigations have demonstrated an association of this variant with age of psychosis onset and schizophrenia symptoms. One recent publication found that the T allele of miR-137 rs1625579 is associated with an earlier age of onset in schizophrenia [17]. In another recent study with a mixed sample of bipolar disorder I, schizoaffective disorder, and schizophrenia patients, the risk variant was also associated with lower positive symptom scores [15]. Green et al. [16] found that the G-allele was associated with a specific schizophrenia phenotype characterized by severe cognitive impairment and negative symptoms. However, these studies did not show a consistent pattern of miR-137 polymorphism with regard to any clinical or neurobiological variables of schizophrenia. Differences in the distribution of allele frequency, diagnostic criteria, symptoms, and cognitive measures may limit the power of these studies to detect association, particularly in case-control studies.

Although the biology underlying the observed association of the miR-137 rs1625579 polymorphism remains unclear, it has been noted that the T allele of rs1625579 may be associated with a decrease in miR-137 expression in the cerebral cortex [27]. miR-137 is likely a central factor coordinating the timing and expression of multiple schizophrenia risk genes, especially those related to neuronal differentiation and proliferation [2, 8, 10, 24, 31, 32]. Thus, we suspect that dysregulation of miR-137 expression and the subsequent abnormal expression of miR-137 target genes contribute to the neurodevelopment, disease progression, and neuromorphological pathologies present in schizophrenia [33].

In conclusion, the results of our case-control study and meta-analysis provide convincing evidence that the miR-137 rs1625579 polymorphism is significantly associated with schizophrenia risk. In addition, the rs1625579 polymorphism modifies working memory performance in Chinese Han patients with schizophrenia. Further investigations on the functional consequences of miR-137 polymorphism may help to elucidate the molecular mechanisms by which this genetic variant affects the schizophrenic phenotype.

## Figures and Tables

**Table 1:** The demographic characteristics of schizophrenic patients and controls.

Variables	Schizophrenic patients	Controls	Statistical tests
	(*n* = 611)	(*n* = 628)	
Mean age ± SD (year)	35.56 ± 13.95	36.54 ± 12.14	*t* = 1.331 *P* = 0.183
Gender *n* (%)			
Male	397 (64.98)	417 (66.40)	*χ* ^2^ = 0.279 *P* = 0.597
Female	214 (35.02)	211 (33.60)	

SD: standard deviation.

**Table 2:** Genotype and allele frequencies of miR-137 gene rs1625579 polymorphism in schizophrenic patients and controls.

	*N*	Genotype *n* (%)		*χ* ^2^	*P*	Allele *n* (%)		*χ* ^2^	*P*	HWE		OR	95% CI
		TT	GT + GG			T	G			*χ* ^2^	*P*		
Total													
Patients	611	550 (90.02)	61 (9.98)	4.409	0.036∗	1159 (94.84)	63 (5.16)	4.971	0.026∗	0.097	0.756	1.450^a^	1.024–2.054
Control	628	541 (86.15)	87 (13.85)			1164 (92.68)	92 (7.32)			0.919	0.338	1.454^b^	1.045–2.024
Male													
Patients	397	353 (88.92)	44 (11.08)	2.275	0.131	748 (94.21)	46 (5.79)	2.562	0.109	0.377	0.539	1.375^a^	0.908–2.081
Control	417	356 (85.37)	61 (14.63)			769 (92.21)	65 (7.79)			0.999	0.317	1.374^b^	0.930–2.032
Female													
Patients	214	197 (92.06)	17 (7.94)	2.240	0.135	411 (96.03)	17 (3.97)	2.548	0.110	0.366	0.545	1.629^a^	0.856–3.099
Control	211	185 (87.68)	26 (12.32)			395 (93.60)	27 (6.40)			0.025	0.876	1.653^b^	0.887–3.079

^a^Calculations were performed TT versus GT + GG; ^b^calculations were performed T versus G; *P < 0.05.

HWE: Hardy-Weinberg equilibrium; OR: odds ratio; 95% CI: 95% confidence interval; nucleotides: G = guanine, T = thymine.

**Table 3:** Analysis of clinical characteristics between patients with TT and GT + GG genotypes.

Variables	*N* (TT/GT + GG)	Genotype		Statistic tests	*P*
		TT	GT + GG		
Age (Mean ± SD, year)	550/61	36.35 ± 14.04	37.41 ± 13.06	*t* = 1.095	0.274
Disease duration (Mean ± SD, year)	550/61	10.43 ± 10.11	10.49 ± 9.38	*t* = 0.045	0.964
Age at onset (Mean ± SD, year)	550/61	24.58 ± 10.24	26.82 ± 10.93	*t* = 1.611	0.108
Male	348/44	23.85 ± 9.01	25.05 ± 9.68	*t* = 0.824	0.411
Female	195/17	26.76 ± 11.14	31.42 ± 12.86	*t* = 1.630	0.105
Family history +: *n* (%)	79/8	79 (90.80)	8 (9.20)	*χ* ^2^ = 0.070	0.791
Family history −: *n* (%)	471/53	471 (89.89)	53 (10.11)		
PANSS (Mean ± SD)					
Total score	533/60	80.16 ± 18.27	79.52 ± 19.30	0.258	0.796
P subscore	533/60	22.45 ± 7.26	21.88 ± 7.53	0.575	0.565
N subscore	533/60	18.33 ± 8.12	19.23 ± 6.95	0.831	0.406
G subscore	533/60	36.83 ± 8.92	36.38 ± 8.94	0.362	0.717
BACS (Mean ± SD)					
Digit sequencing task	185/24	15.58 ± 9.56	19.71 ± 8.18	2.016	0.045∗
Category instances	185/24	30.92 ± 12.67	31.79 ± 12.19	0.317	0.752
Controlled oral word association test	185/24	10.04 ± 6.07	10.04 ± 5.21	0.001	0.999
List learning	185/24	22.29 ± 12.61	21.33 ± 13.93	0.346	0.730
Token motor task	185/24	46.14 ± 11.83	46.29 ± 12.64	0.061	0.952
Tower of London	185/24	7.62 ± 6.85	7.50 ± 1.12	0.083	0.934
Symbol coding	185/24	21.12 ± 12.68	18.71 ± 12.31	0.881	0.379

*P < 0.05.

SD: standard deviation; PANSS: Positive and Negative Syndrome Scale; P: Positive scale; N: Negative scale; G: General Psychopathology scale; BACS: Brief Assessment of Cognition in Schizophrenia.
